# Molecular Dynamics Simulations of Molecular Diffusion Equilibrium and Breakdown Mechanism of Oil-Impregnated Pressboard with Water Impurity

**DOI:** 10.3390/polym10111274

**Published:** 2018-11-16

**Authors:** Yi Guan, Ming-He Chi, Wei-Feng Sun, Qing-Guo Chen, Xin-Lao Wei

**Affiliations:** 1 Heilongjiang Provincial Key Laboratory of Dielectric Engineering, School of Electrical and Electronic Engineering, Harbin University of Science and Technology, Harbin 150080, China; yiguan_hrbust@126.com (Y.G.); minghe_chi@126.com (M.-H.C.); qgchen_hrbust@126.com (Q.-G.C.); xinlao_wei@126.com (X.-L.W.); 2 Key Laboratory of Engineering Dielectrics and Its Application, Ministry of Education, Harbin University of Science and Technology, Harbin 150080, China

**Keywords:** oil-impregnated pressboard, molecular dynamics simulation, phase equilibrium, electric breakdown

## Abstract

The water molecule migration and aggregation behaviors in oil-impregnated pressboard are investigated by molecular dynamics simulations in combination with Monte Carlo molecular simulation technique. The free energy and phase diagram of H_2_O-dodecylbenzene (DDB) and H_2_O-cellulose mixtures are calculated by Monte Carlo technique combined with the modified Flory-Huggins model, demonstrating that H_2_O molecules can hardly dissolved with infinitesimal content in cellulose system at temperature lower than 650 K, based on which the oil/cellulose layered structure with water impurity representing three-phase coexistence in oil-impregnated pressboard are modeled and performed for molecular dynamics. The molecular dynamics of H_2_O/DDB/cellulose three-phase mixture simulating oil-paper insulating system with H_2_O impurity indicates that DDB molecules can thermally intrude into the cellulose-water interface so as to separate the water phase and cellulose fiber. The first-principles electronic structure calculations for local region of H_2_O/DDB interface show that H_2_O molecules can introduce bound states to trap electrons and acquire negative charges, so that they will obtain sufficient energy from applied electric field to break DDB molecular chain by collision, which are verified by subsequent molecular dynamics simulations of H_2_O^−^/DDB interface model. The electric breakdown mechanism under higher than 100 kV/m electric field is presented based on the further first-principles calculations of the produced carbonized fragments being dissolved and diffusing in DDB phase. The resulted broken DDB fragments will introduce impurity band between valence and conduction bands of DDB system, evidently decreasing bandgap as to that of conducting materials in their existence space. The conductance channel of these carbonized DDB fragments will eventually be formed to initiate the avalanche breakdown process by the cycle-feedback of injected charge carriers with carbonized channels.

## 1. Introduction

Synthetic liquid insulating material such as synthetic oil is a mixture of many hydrocarbons, the purification and preparation process of which is complex. Because the general insulating oil is easy to burn and has low heat resistance and dielectric constant, it is necessary to study and develop novel kinds of liquid insulating materials with excellent properties [1]. Therefore, the formation mechanism of dielectric properties of liquid insulating materials should be adequately understood. Under the condition of transformer operation, the insulating oil is inevitably dissolved with trace impurities (such as water), which determine the breakdown characteristics of the insulating oil by the distribution and state of these impurities [2]. Therefore, the breakdown characteristics of oil insulation mainly depend on the impurity state and content in microsoluble insulating oil [3]. Transformer oil is a product of petroleum fractionation, the main components of which are alkanes, naphthenic saturated hydrocarbons, aromatic unsaturated hydrocarbons, and other compounds. The predominant component of mineral oil used as insulation dielectrics in transformer consists of multiple dodecylbenzene (DDB) molecules. DDB is primarily applied in impregnated-paper or paper-film composite medium which can be used in cables, capacitors, and transformers [4]. DDB is the dominant liquid insulating material of oil-paper insulation medium in high-voltage transformers. DDB is a mixture of alkylbenzene with 9~15 carbon atoms in side chain which is called rigid or soft alkylbenzene, respectively, when the side chain has a branched chain that is not biodegradable or when the side chain is straight that is easy to biodegrade. DDB is a weak polar material with good electrical properties and thermal, oxygen aging stability, good inspiratory property, high breakdown electric field strength. Furthermore, the copper, steel, zinc, tin, aluminum, and other metals have little catalytic aging effect on DDB [5]. Water molecules are polar molecules and are the main impurities in the synthesis and application of insulating oils [6]. The purity of DDB preparation and extraction is higher than that of other liquid insulating materials, but it is still difficult to remove all moisture. Thus, water impurity plays a key role in the dielectric properties and breakdown process of DDB.

At present, the current experimental studies on the breakdown characteristics of oil–paper composite insulation in transformer cannot obtain enough evidence to explain the breakdown electric field phenomenon in the working range at room temperature, and the oil-insulating mechanism is still in the stage of speculation [7,8]. It is urgent to provide theoretical basis for microscopic mechanism by special schemes to study on molecular/atomic scale. Molecular dynamics (MD) simulation based on atomic force field and dynamic algorithm can more accurately simulate the transition of a large number of molecular systems from non-equilibrium states to thermodynamic states at atomic scale [9]. The structure and properties of the molecular and material system at non-zero temperature can be predicted by the molecular dynamics simulation method based on molecular and crystal database, combined with molecular mechanics, quantum mechanics, and molecular graphics technology, which is a very important method in the field of material modification and design. Although it is difficult to get a clear understanding on the electric breakdown mechanism of insulating oil impregnated in pressboard from experiments, the correlated molecular dynamics studies of properties and mechanisms have not been reported until now. In this paper, the dissolution and mixing state of water molecules in (DDB) liquid immersed by pressboard for actual transformer insulation are simulated by molecular dynamics at different temperatures and electric fields, and the microstructure, molecular diffusion, phase equilibrium and local electronic structure of oil-impregnated pressboard with trace water are calculated to explore the underlying mechanism of electric breakdown induced by water impurity.

## 2. Theoretical Methodology

**Figure 1:**
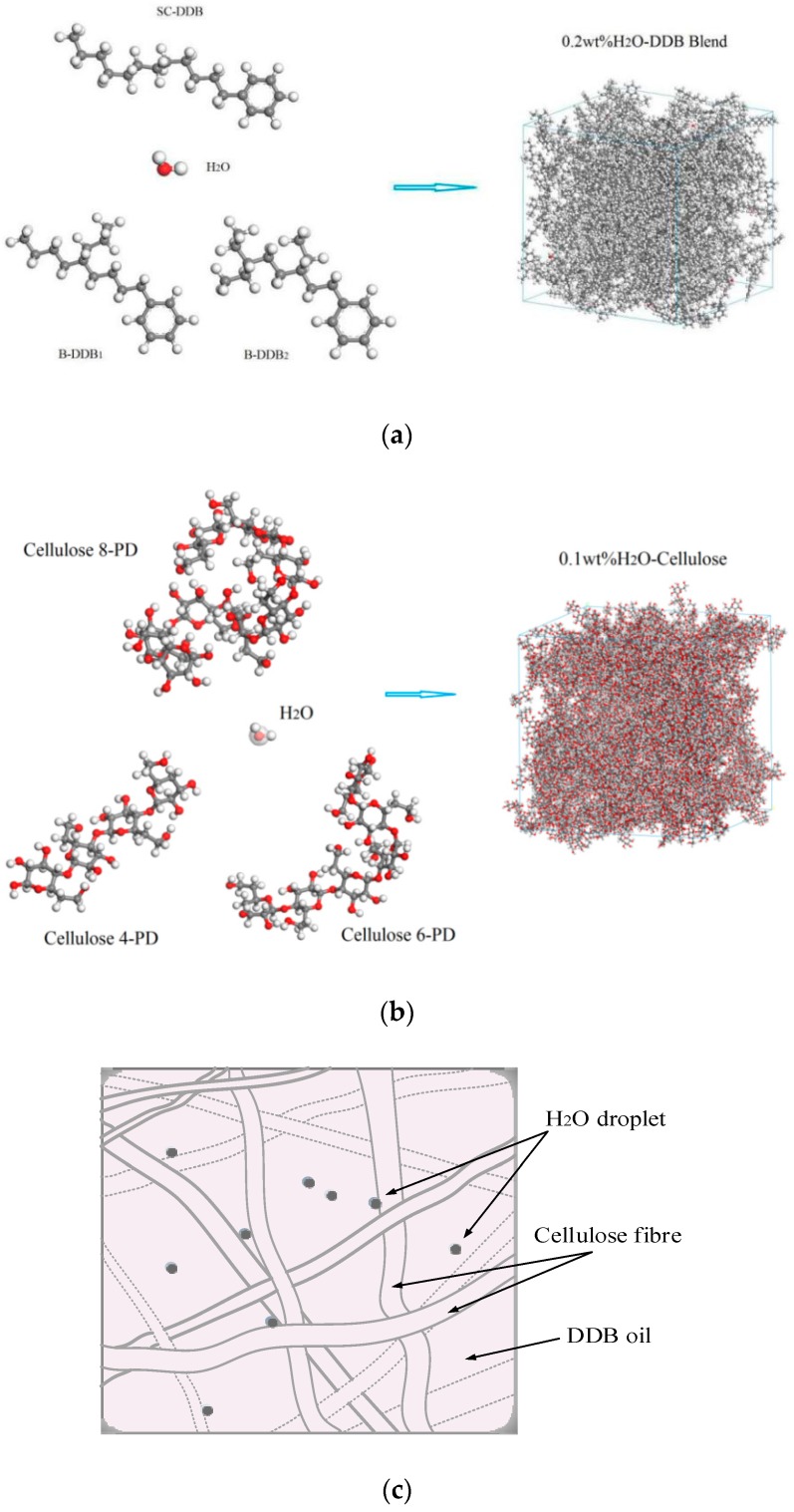
Molecular models for amorphous multi-molecules mixed systems constructed from solute H_2_O with (**a**) three types of DDB molecule and (**b**) three pieces of cellulose molecules in 4, 6, and 8 polymerization degrees. Gray, white, and red balls represent carbon, hydrogen, and oxygen atoms respectively; (**c**) Schematic microstructure of porous intervals between cellulose fibers with H_2_O molecule cluster (water impurity) absorbed on them in pressboard.

The blend models for multiple molecules systems of H_2_O-DDB and H_2_O-cellulose mixtures with the densities of 0.855 g/cm^3^ and 1.430 g/cm^3^ respectively are constructed by Monte Carlo procedure as implemented by Amorphous Cell code of Materials Studio 8.0 software package (Accelrys Software Inc., San Diego, CA, USA) [10,11,12], before and after which the geometry optimization (energy minimization) for initial H_2_O, DDB, cellulose molecules and the molecular dynamics simulations for mixing systems are respectively performed employing by the Forcite code of Materials Studio. Both the simulated mixtures consist of three different DDB or cellulose molecules respectively to model approach-to-real polymer materials, as shown in Figure 1 illustrating the molecular constituents in molecular-mixing amorphous cell for 0.3 mol % H_2_O-DDB and 0.5 mol % H_2_O-cellulose mixtures. Employing the rotational isomeric state (RIS) model [13], the simulated cellulose molecules with tacticity and random torsion are constructed from glucose monomers to be connected by glycosidic bonds in 5–10 polymerization degrees, as shown in Figure 1b, with the consideration that the cellulose molecules are in very low polymerization degree in amorphous regions of insulation pressboard where the H_2_O molecules can possibly dissolved [14].

**Figure 2:**
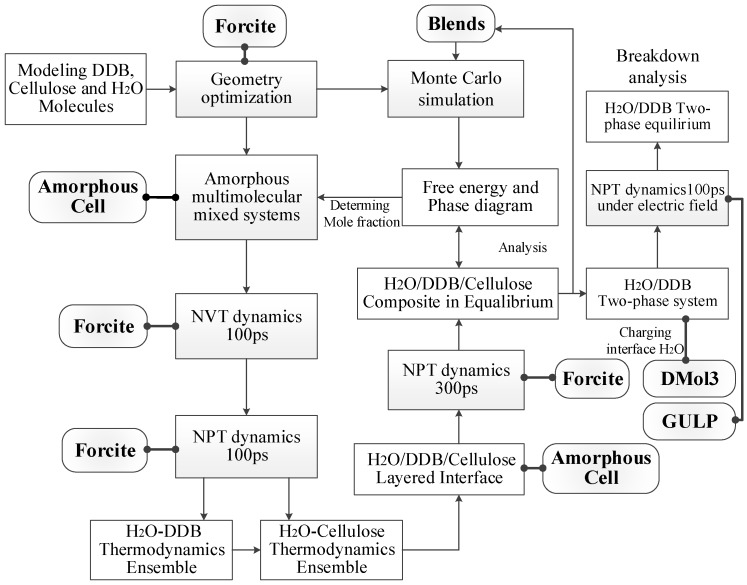
Schematic procedure of modeling (Amorphous cell), Monte Carlo (Blends), and molecular dynamics (Forcite and GULP (General Utility Lattice Program)) simulations, first-principles calculation (DMol3).

Molecular interaction binding energy, free energy, and phase diagram of H_2_O–DDB and H_2_O–cellulose mixtures are calculated by Monte Carlo molecular simulation technique combined with the modified Flory-Huggins model, as implemented in Blends module of Materials Studio 8.0 package, to investigate the compatibility of H_2_O in DDB and cellulose [15,16]. The molecular dynamics simulations as implemented in Materials Studio 8.0 Forcite code are performed for H_2_O–DDB, H_2_O–cellulose mixtures to obtain equilibrium structures and for H_2_O/DDB/cellulose three-phase system to investigate the H_2_O transport and aggregation at the interface of mineral oil and cellulose in oil-impregnated pressboard. The modeled H_2_O/DDB/cellulose three-phase system for oil-impregnated pressboard insulation are constituted by packing tool of Amorphous Cell code, as shown in Figure 1c, based on the pre-constructed DDB–cellulose double layer structure with layer thickness ratio of approximate 1:1, and interface thickness of 5Å which is larger and shorter than chemical bonding and van der Waals interaction distances. The Amorphous Cell calculations have performed a geometry optimization, but the constructed initial structures may still contain stress points and density inhomogeneities that need to be relaxed dynamically. The procedure from molecule and mixture model to free energy/phase diagram calculation and molecular dynamics simulation is schematically exhibited in Figure 2. 

The geometry optimization for atomic structure and MD simulations are carried out with the PCFF (polymer consistent forcefield) atomic force field which has been verified to be valid for a majority of polymers and compounds [17]. The PCFF has been developed from an earlier forcefield CFF91 intending to be utilized for polymers, organic and inorganic materials, demonstrated to be qualified for such as melamine resins, polycarbonates, polysaccharides and about 20 inorganic metals, as well as for carbohydrates, lipids and nucleic acids. PCFF can be effectively used to calculate mechanical property, cohesive energy, compressibility, elastic constants and heat capacity. The detailed scheme and parameter setup adopted for geometry optimization and MD simulations are listed in the Table 1. The time step of dynamics taken to sufficiently obtain numerical stability depends on the maximum frequency of atomic vibration formalized with dynamics equation and the corresponding integration algorithms. It can be reasonably assumed the velocity and acceleration being constant in 1/15–1/20 of vibration period, which complies to the common rule-of-thumb that the maximum frequency vibration should be sampled between 15 and 20 times through one cycle. The highest vibration frequency in the carbon-based system originates from the stretching C–H bonds of ~10 fs. Hence the time step should be set as 0.5–0.7 fs. For the amorphous system consisting of large polymer molecules, the MD simulation of NPT ensemble will approach thermodynamic equilibrium after 10 ps; while for the composite nanolayered structure to adequately stabilize molecule diffusion at interface and relax interfacial structure, the total simulation time is required to be set substantially longer. Therefore, the time step and total simulation time are set as 0.5 fs and 100.0 ps respectively to acquire definite thermodynamic ensemble and accurate dynamics calculations.

## 3. Results and Discussion

### 3.1. Free Energy and Phase Diagram

The best known thermodynamics theory of mixing and phase separation in binary systems is the Flory-Huggins model [15,16], in which the free energy for mixing a binary system is represented as:

(1) $$\frac{\Delta G}{RT}=\frac{\varphi_{b}}{n_{b}}\ln\varphi_{b}+\frac{\varphi_{s}}{n_{s}}\ln\varphi_{s}+\chi \varphi_{b}\varphi_{s}$$

where Δ*G* denotes the free energy of mixing (per mole), *φ*_i_ symbolizes the volume fraction of component i, *n*_i_ represents the degree of polymerization of component i, *χ* is the interaction parameter, *R* and *T* identify gas constant and absolute temperature respectively. The first two terms represent the combinatorial entropy, being always negative and thus preferring a mixed state over pure components, while the last term characterizes the free energy from interaction, deprecating a mixed state if the interaction parameter *χ* is positive. The balance between the two contributions gives rise to various phase diagrams. Phase diagrams are useful in illustrating the compatibility of binary mixtures. A phase diagram generally contains three pieces of information: critical points, binodals and spinodals. At the critical point, both the second derivative and third derivatives of the free energy with respect to composition vanish. The coexistence region is bound by the binodal (the blue line in Figure 4), in which the mixture can lower its free energy by separating into two phases. The coexistence region also gives rise to two compositions where the second derivative of the free energy is zero. The line through those points is called the spinodal. The spinodal separates the coexistence region into two regions: the mixture is metastable only start phase separating after a sufficiently large fluctuation between the binodal and spinodal. The mixture is unstable in the region bounded by the spinodal, and thus any fluctuation will cause the mixture to spontaneously phase separate. 

**Figure 3:**
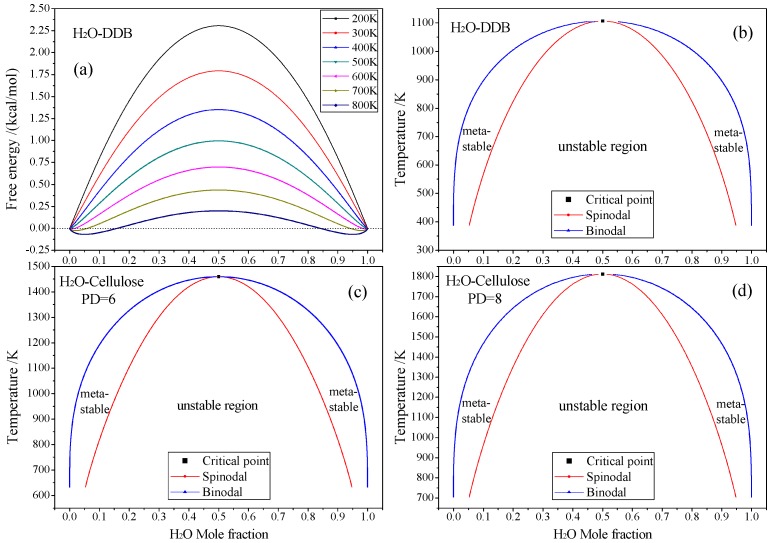
(**a**) The free energy of mixing for H_2_O-DDB mixture varying with Mole fraction for 200–800 K temperatures, mixture phase diagrams of (**b**) H_2_O-DDB; (**c**) H_2_O-Cellulose (PD = 6) and (**d**) H_2_O-Cellulose (PD = 8).

According to Equation (1), the increment of polymerization degree generally decreases miscibility, since the energy term (prefer to phase separation) increases with respect to the entropy term (favoring mixing). Therefore, applying for H_2_O molecules solute in amorphous cellulose system, the H_2_O solubility decreases appreciably with polymerization degree of cellulose chains, as the free energy and phase diagram results shown in Figure 3 from our calculations. These results are inconsistent with the verdict concluded by K. Mazeau that no evident variation has been found for physico-chemical properties of cellulose when length of molecular chain varies [14]. Although H_2_O solubility in DDB and cellulose are pretty small, there are substantial metastable regions between spinodal and binodals in phase diagram presenting significantly higher H_2_O molecule concentrations approaching to 5% in Mole fraction, as illustrated in Figure 3. These results predict there may exist about 0.4 wt. % metastable H_2_O molecules in DDB and amorphous cellulose system at 400 K and 650 K respectively, based on which for the subsequent molecular dynamics simulation, we construct unsaturated mixture model with Mole percentage under these values determined from calculated phase diagram.

Base on the experiments, the substantial water constituents can been absorbed into pressboard, while H_2_O molecules cannot saluted with nearly neglected content in cellulose system at temperature lower than 650 K from our Monte Carlo calculation results as shown in Figure 3c,d. This is due to that the H_2_O absorbed in the pressboard actually aggregate in the pores formed between cellulose fibers without permeating into the fibers constructed from cellulose molecules, as illustrated from Figure 1c.

### 3.2. Diffusion and Phase Equilibrium

Although H_2_O molecule densities in the DDB and cellulose are appreciably different, when the two equilibrium phases of DDB and cellulose are contacted to each other, H_2_O molecules in cellulose with much lower density could diffuse towards DDB side because that it is the interactions from the based solvent of DDB or cellulose, not the random collisions between H_2_O molecules, determining the molecule migration of solute H_2_O. The fugacity of solute component in solution or mixture characterizes the solute-solvent molecular interaction and thus the pressure deriving from molecule diffusion of solute, therefore the H_2_O diffusion trend at DDB/cellulose interface lies thermodynamically on the individual H_2_O fugacities in DDB and cellulose. Another thermodynamic element that may dominate the H_2_O migration at the DDB/cellulose interface is interface free energy which in thermodynamics identify the interaction between DDB and cellulose molecules at interface. It is also definitely noted from the molecular dynamics simulation that the H_2_O molecules cannot dissolve or permeate into the cellulose phase so that the H_2_O molecules have been excluded from the cellulose system in the completed modeling of H_2_O/DDB/cellulose three-phase coexistence.

**Figure 4:**
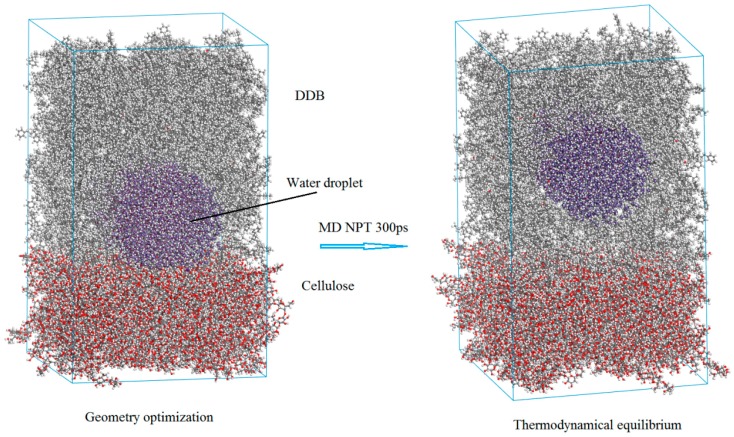
The initial geometrically optimized configuration (**left**) and phase equilibrium (**right**) of modeled H_2_O/DDB/cellulose 3-phase system for oil-impregnated pressboard.

The H_2_O/DDB/cellulose three-phase mixture system is firstly constructed by multi-molecules Monte Carlo method of packing technique as implemented by Amorphous Cell program, in which the atomic geometry has also been optimized. Then the initially modeled configuration is thermodynamically equilibrated by performing molecular dynamics simulation to represent oil-paper insulating system containing H_2_O impurity at ambient temperature and pressure. In practical transformer insulation, before pressboard being impregnated in DDB to form oil-paper complex dielectrics, the condensed H_2_O cluster have absorbed on cellulose fibers in porous intervals of pressboard even after heating treatment in vacuum drying oven. Therefore, in the practical process of preparing oil (DDB)-impregnated pressboard, the DDB permeate into the micro-pores as interval space between cellulose fibers of pressboard, and clad cellulose fibers together with H_2_O condensed cluster absorbed on them, as initial three-phase model shown in left Figure 4. Because of the relatively better miscibility of DDB-cellulose than that of cellulose-water, under the thermodynamic driving forces from interface free energies (interface tensions) of three coupling interfaces, the cellulose-water interface spontaneously shrinks and finally disappears, which exactly means the DDB molecules thermally diffuse as intruding into the cellulose-water interface and eventually separate the water phase and cellulose fiber, as shown in right Figure 4 of phase equilibrium after thermodynamics simulation.

### 3.3. Electric Breakdown Process

**Figure 5:**
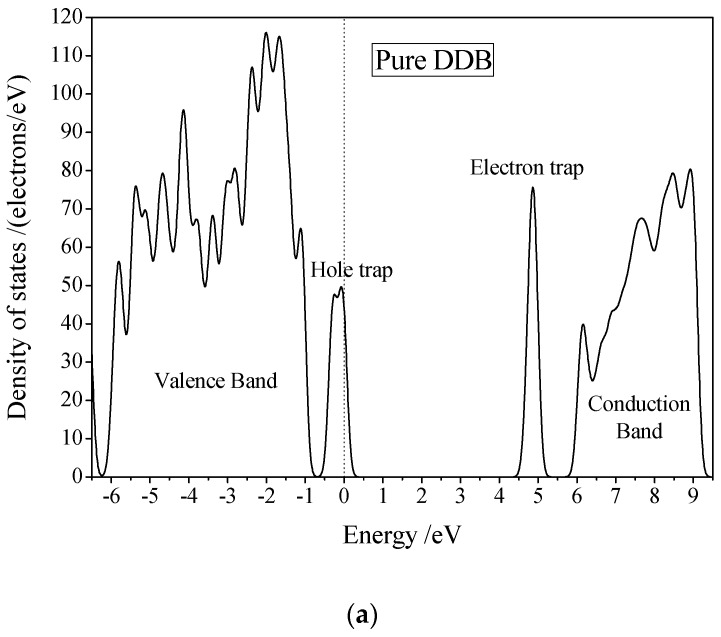

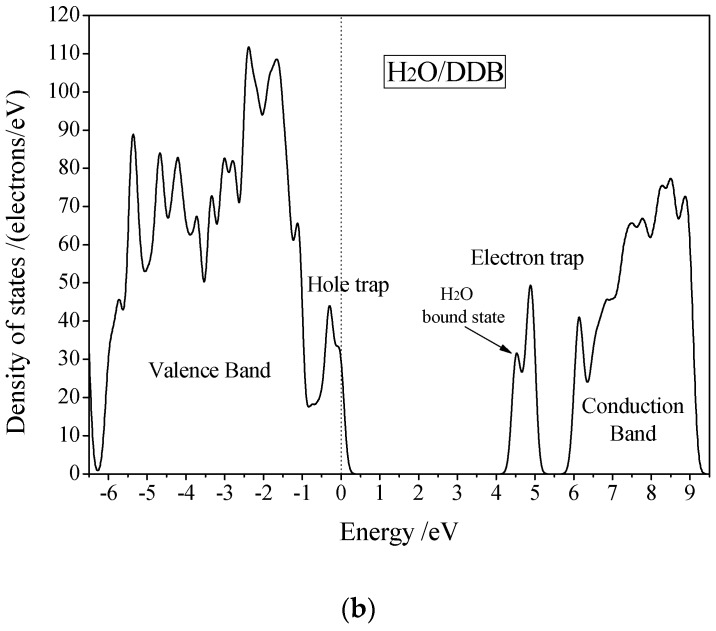
Electronic energy density of ground state for (**a**) pure DDB and (**b**) H_2_O/DDB mixed system (local region at two-phase interface) with the highest occupied state as reference energy zero, Gauss smearing is set as 0.1 eV.

In order to determine the charge distribution and the motion of H_2_O molecule under the electric field, the electronic structure and charge distribution of the simplified model of H_2_O/DDB mixture (including six DDB molecules and one water molecule, representing the local region of the H_2_O/DDB two-phase interface) under different temperatures and electric fields are calculated by all-electron numerical orbital first-principles method with PBEsol form of GGA correlation-exchanged functional as implemented in DMol3 code [20]. The localized trap state introduced by H_2_O molecule and its electrical transport behavior in DDB are analyzed based on the energy density of states (DOS), as the calculated results shown in Figure 5. Calculation of Mulliken population charge of H_2_O (sum of Mulliken charges of hydrogen and oxygen atoms) is calculated from self-consistent charge density. The calculated results of DOS indicate that the unoccupied bound state (the wave function is localized near the H_2_O molecule) introduced by H_2_O in the band gap of DDB is 0.5 eV lower than the intrinsic electron trap level of DDB matrix which is about 1.0 eV lower than conduction band minimum, as shown in Figure 5b. This bound state can also be looked as the deeper trap state for the electrons of conduction band, which is energetically preferable for electron occupation and will move together with H_2_O thermal diffusion. Meanwhile, the water molecule and the DDB matrix molecule interact by van der Waals force without electronic bonding. Therefore, it is necessary to leap over the energy barrier between H_2_O and DDB molecules which turns to be lowest in the thermal collision between H_2_O and DDB molecules at H_2_O/DDB interface. Consequently, the electrons in conduction band or intrinsic trap states of DDB phase will trap into the unoccupied bound state of H_2_O molecule so as to make H_2_O being loaded with negative charge after the molecular collision, and more hole will be produced simultaneously. The frequency of collision between H_2_O and DDB due to thermal motion increases with temperature increment. Thus, the probability of producing negatively charged H_2_O ion from electron transfer of trapping transition will be raised when temperature goes up. Population analysis of Mulliken atomic charge confirms that the charge on H_2_O is negative, hence the H_2_O^−^ ion produced by thermal collision and electron transfer will transport in the electric field as net motion in the inverse direction of electric field.

The molecular dynamics simulation utilizes the semi-empirical force field with assigning immutable charge to individual atoms, which cannot reflect the electron transfer process as calculated by the first principles kinetics. However, the first principles calculation is limited by the computational cost, which cannot be used to calculate the whole multi-phases model. In actual electric breakdown experiments, the electrodes are applied in contact with the DDB oil, and the electrons transferred to H_2_O molecule actually come from the electrode-injected charge carriers rather than a small number of thermally excited electrons. Therefore, in the molecular dynamics simulation, the assigning first-principles calculated charges to H_2_O molecules can truly represent the real situation of the charge injection by the external electrodes in experiments. Accordingly, The H_2_O/DDB two-layer model is constructed to simulate the interface of water/oil two-phase coexistence system, in which H_2_O molecules on H_2_O/DDB two-phase interface are assigned by charge equivalent to first principles calculated Mulliken charge (the positive charge of the matrix is negligible), and the molecular dynamics simulations with ReaxFF 6.0 force field are performed at ambient temperature and under high electric field with the direction along two-phase interface, as implemented by General Utility Lattice Program (GULP) of materials studio 8.0 package [21].

**Figure 6:**
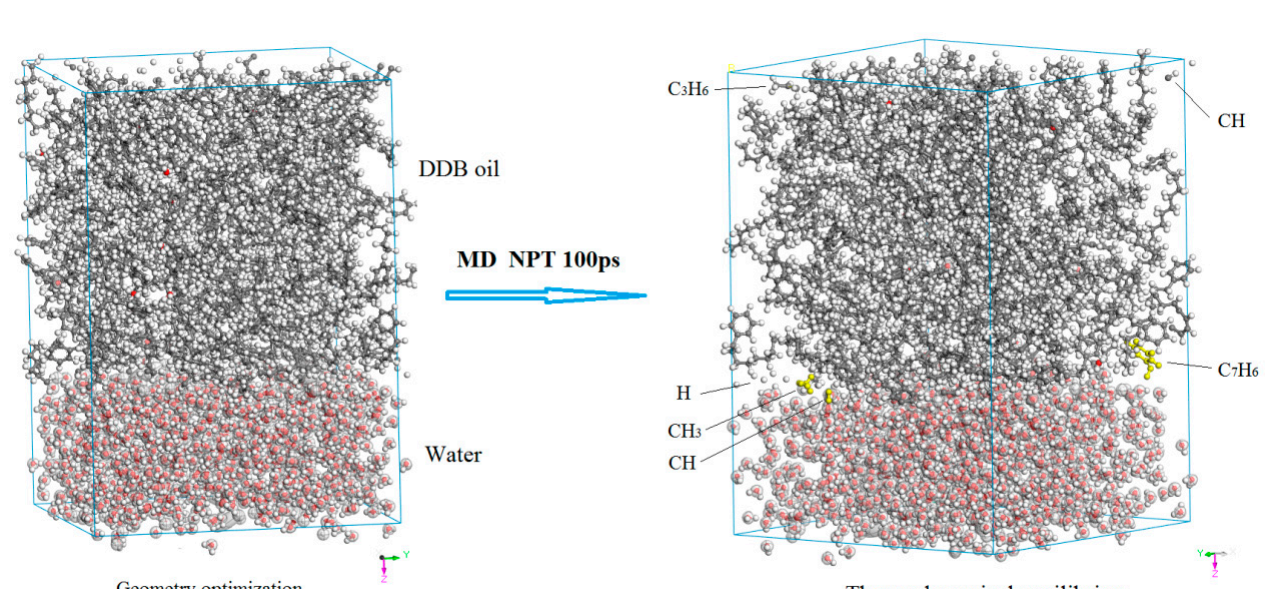
Morphological structures of H_2_O^−^/DDB two-phase system at 300 K temperature and 200 kV/m electric field before and after reaching thermodynamic equilibrium from molecular dynamics simulation.

**Figure 7:**
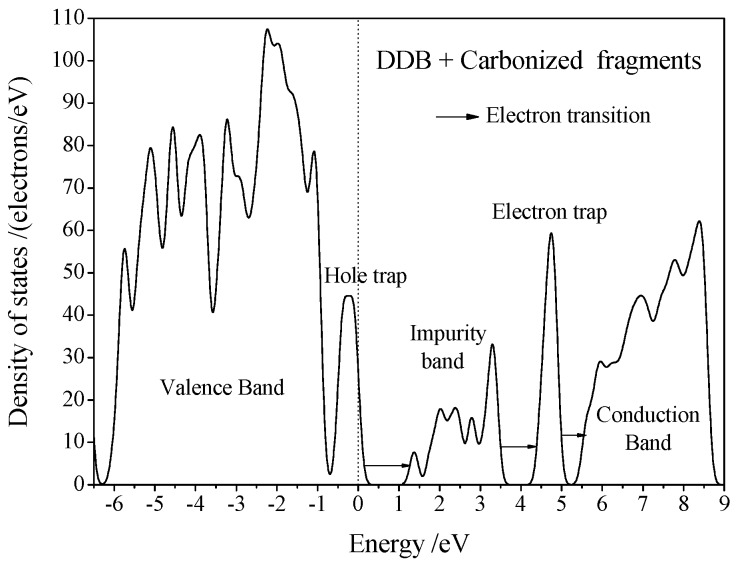
Electronic density of states for mixture system containing DDB molecules and CH, CH_3_, C_3_H_5_, and C_7_H_6_ unsaturated broken fragments of DDB molecular chain, with the highest occupied state as reference energy zero, Gauss smearing is set as 0.1 eV.

The molecular dynamics simulations of H_2_O^−^/DDB two-phase system under high electric field indicate that when the electric field is high enough (>100 kV/m), the ambient temperature collisions between H_2_O with DDB molecules at two-phase interface of oil-water in transformer insulating oil will lead to the breaking of the DDB molecular chain and the formation of CH, CH_3_, C_3_H_5_, C_7_H_6_, and other unsaturated carbonic fragments which will diffuse into the DDB liquid phase. Figure 6 exhibits structural result of molecular dynamics simulation for H_2_O^−^/DDB two-phase system at 300 K temperature and 200 kV/m electric field, in comparison with the geometrically optimized structure without atomic kinetics and electric field (before thermodynamic equilibrium). According to the first principles electronic structure calculations, the molecular orbital energy levels of these fracture fragments are just located in the DDB band-gap, as shown in Figure 7. Therefore, under the extremely high electric field, H_2_O^−^ ions can obtain high enough kinetic energy as to break C–C and C–H bonds of DDB molecular chain when they collide with DDB molecules. The resulted broken DDB fragments as described by carbonized DDB constituents will introduce impurity band between valence and conduction bands of DDB matrix, and thus cause bandgap to decrease lower than *kT* (*k* is Boltzmann constant, *T* denotes temperature) as that of conducting materials in their existence space. With these carbonized DDB constituents being continuously produced at the water/oil interface under high electric field and diffusing through the DDB oil, the conductance channels constituted by high density of them will been constructed. In the actual electric breakdown experiment, the electrodes will be applied at two terminals of oil-impregnated pressboard in direct contact with DDB oil, and after the conductance channel penetrates the oil, the avalanche breakdown process will be initiated: a large number of carriers are injected from both electrodes and obtain sufficient kinetic energy under the action of high electric field so as to break C–C or C–H bonds of DDB molecules and sharply increase the density and region of carbonized DDB constituents, which as a feedback aggravates the carrier injections by conductance channel expansion to the surrounding area. The formation of “avalanche” similar to the discharge process results in the ultimate damage of DDB dielectrics.

## 4. Conclusions

Employing molecular dynamics simulations in combination with Monte Carlo technique and first-principles calculations, the H_2_O molecule diffusion and aggregation at interfaces of H_2_O/DDB/cellulose three-phase coexistence in oil-impregnated pressboard with water impurity are simulated to investigate electric breakdown mechanism of oil-paper composite insulation utilized in high voltage transformers. The phase diagrams of H_2_O–DDB and H_2_O–cellulose mixtures from Monte Carlo molecular simulations indicate that H_2_O molecules cannot dissolved into cellulose system at temperature lower than 650 K, attributed to which the layered three-phase coexistence system of oil/cellulose interface structure with water impurity are modeled with molecular dynamics. The molecular dynamics of H_2_O/DDB/cellulose three-phase dominating the breakdown process of oil-paper insulating system containing H_2_O impurity demonstrate that DDB molecules will diffuse thermally through cellulose-water interface, resulting in the separation of water phase and cellulose fiber. The subsequent first-principles electronic structure calculations for H_2_O/DDB interface region prove that H_2_O molecules can acquire negative charges by trapping electrons into their bound states through molecular collisions and will obtain adequate energy from electric field to fracture DDB molecular chain by collision, which are confirmed by correlated molecular dynamics simulations of H_2_O^−^/DDB interface model. The electric breakdown mechanism under higher than 100 kV/m electric field is revealed theoretically based on the further first-principles calculation of the carbonized fragments resulted from breaking DDB molecules by high-electric-field-triggered violent H_2_O^−^ collisions. The produced carbonized DDB fractures will introduce assistant band between valence and conduction bands of DDB matrix, which decreases bandgap in their existence space to the value as that of conducting material. It is reasonably predicted that the conductance channel constituted by these continuously condensed and extended carbonized regions will be promptly formed so as to establish the avalanche breakdown process through cycle-feedback of injected charge carriers with carbonized channels under high voltage.

## Figures and Tables

**Table 1:** Methodology and parameters employed in geometry optimization and molecular dynamics simulation.

Topics	Setting up	Methodology	Condition and Parameters
Energy	Forcefield	PCFF	
	Electrostatic and van der Waals	Atom based summation	Cutoff distance: 18.50 Å Buffer width: 0.5 Å
Geometry optimization	Optimization algorithm	Smart	Convergence:1 × 10^−5^ kcal/mol Maximum iterations: 2 × 10^4^
	Ensemble	NVT	Constant volume/constant temperature
Molecular dynamics	Ensemble	NPT	Constant pressure/constant temperature
	Thermostatic control	Nosé-Hoover-Langevin (NHL) algorithm [18]	Temperature: 300 K, 400 K
	Barostatic control	Souza-Martins algorithm [19]	Pressure: 1 × 10^5^ Pa
	Time integration	Total simulation time	100.0 ps
		Time step and number	0.5 fs and 2 × 10^5^
	Simulation step control	Integration tolerance	Energy deviation: 5000.0 kcal/mol
